# Palliative patients under anaesthesiological care: a single-centre retrospective study on incidence, demographics and outcome

**DOI:** 10.1186/s12871-015-0143-4

**Published:** 2015-11-13

**Authors:** Christoph L. Lassen, Susanne Aberle, Nicole Lindenberg, Annika Bundscherer, Tobias W. Klier, Bernhard M. Graf, Christoph H. Wiese

**Affiliations:** 1 Department of Anaesthesiology, University Hospital of Regensburg, Franz-Josef-Strauss-Allee 11, D-93053 Regensburg, Germany; 2 Department of Nuclear Medicine, University Hospital of Zurich, Raemistrasse, Zurich, Switzerland

**Keywords:** Palliative care, Anesthesiology, Operating rooms, Perioperative period

## Abstract

**Background:**

While anesthesiologist’s involvement in palliative care has been widely researched, extensive data on palliative patients under anesthesiological care in the operating room is missing. This study was performed to assess the incidence, demographics, and outcome of palliative patients under anesthesiological care.

**Methods:**

We conducted a single-center retrospective chart review of all palliative patients under anesthesiological care at a university hospital in 1 year. Patients were classified as palliative if they fulfilled all predefined criteria (a) incurable, life-threatening disease, (b) progression of the disease despite therapy, (c) advanced stage of the disease with limited life-expectancy, (d) receiving or being in need of a specific palliative therapy. Demographics, periprocedural parameters, symptoms at evaluation, and outcome were determined using different medical records.

**Results:**

Of 17,580 patients examined, 276 could be classified as palliative patients (1.57 %). Most contacts with palliative patients occurred in the operating room (68.5 %). In comparison to the non-palliative patients, procedures in palliative patients were significantly more often urgent or emergency procedures (39.1 % vs. 27.1 %., *P* < 0.001), and hospital mortality was higher (18.8 % vs. 5.0 %, *P* < 0.001). Preprocedural symptoms varied, with pain, gastrointestinal, and nutritional problems being the most prevalent.

**Conclusions:**

Palliative patients are treated by anesthesiologists under varying circumstances. Anesthesiologists need to identify these patients and need to be aware of their characteristics to adequately attend to them during the periprocedural period.

## Background

Anaesthesiologists have been involved in the care of palliative patients since the beginning of modern palliative medicine. The field of anaesthesiology covers many parts of palliative medicine, e.g. pain management, use of sedative/anxiolytic medication, and treatment of critically ill patients. Also palliative medicine and anaesthesiology are similar to each other as both view integrating and coordinating therapies of different medical specialties as an important aspect of their specialty (e.g. physical and social therapists in pain management, spiritual care in the intensive care unit). Therefore some authors see anaesthesiologists as ideal physicians for the care of palliative patients [1, 2].

Besides the anaesthesiologist’s involvement in palliative medicine, palliative patients are also treated by anaesthesiologists in the intensive care unit [3], in pain management [4], and in pre-hospital emergency medicine [5]. Management of palliative patients in the anaesthesiologist’s primary workplace, the operating room, has not been in the immediate focus of the literature. Most articles on palliative patients in the operating room have been written from a surgical perspective with an emphasis on indication of surgical treatment, types of palliative surgical procedures, and surgical outcome [6, 7]. Anaesthesiological literature on the other hand has focused on describing the ethical problems in the care for palliative patients with do-not-resuscitate-orders in the perioperative period [8, 9]. Only few articles describe the perioperative medical management of palliative patients [10]. While incidences of palliative surgeries have been described in the literature [11, 12], no data to our knowledge exists on the incidence of palliative patients being under the care of an anaesthesiologist in the operating room or during other procedures.

Our study was undertaken to find out how often anaesthesiologists treat palliative patients during the periprocedural period and to describe the demographics, the anaesthesiological treatment, the medical problems, and the outcome of this patient group.

## Methods

### Study setting, population, data collection

We conducted this single-centre, retrospective study at the University Hospital of Regensburg (Regensburg, Germany) following ethical approval (Ethical Committee of the University Hospital of Regensburg, protocol number 10-101-0248, approved 12/14/2010). The requirement for individual informed consent was waived. Before the final analysis, we removed all identifiable data from the dataset except age and sex.

We examined all patients under anaesthesiological care at our university hospital during a 1-year period (01/01/2009-12/31/2009). This included patients scheduled for operations, interventional and diagnostic procedures. For a better comprehensibility, we use the term procedure for operations as well as for all types of interventional and diagnostic care in this article. In patients with multiple procedures, each procedure was counted as a new patient contact, since for every procedure a new anaesthesiological evaluation was performed. Again, for a better comprehensibility, we use the term patient throughout this article for each new patient contact.

We obtained all diagnoses at hospital discharge through the electronical hospital medical record database encoded in the current International Statistical Classification of Diseases and Related Health Problems (ICD-10). In our first analysis we included the patients who had a diagnosis of a potentially life-limiting disease as shown in Table 1. Of these patients all relevant medical records (e.g. discharge letters, procedure protocols, and anaesthesia protocols) were screened to obtain the primary intent of therapy. Patients who were treated with a curative intent (e.g. complete tumour resection, receiving adjuvant chemotherapy) were excluded. The remaining patients were defined as palliative patients if they fulfilled all of the following criteria at the time of preprocedural anaesthesiological evaluation, (a) incurable, life-threatening disease, (b) progression of the disease despite therapy, (c) advanced stage of the disease with limited life-expectancy, (d) receiving or being in need of a specific palliative therapy to alleviate symptoms. These criteria are based on a review on how to define a palliative care patient [13].

**Table 1:** ICD-10 Diagnoses that are classified as potentially life-limiting diseases

Disease entity	ICD-10 code
Malignant neoplasms	C00-C97
Neurological diseases	
Systemic atrophies	G10-G14
Persistent vegetative state	G93.8
Dementia	F00-F03
AIDS	B22
Advanced COPD (Gold classification IV)	J44.00, J44.10, J44.80, J44.90
Advanced CHF (NYHA classification IV)	I50.14

*ICD-10* International Statistical Classification of Diseases and Related Health Problems, *AIDS* Acquired immunodeficiency syndrome, *COPD* Chronic obstructive pulmonary disease, *CHF* Chronic heart failure, *NYHA* New York Heart Association

We electronically and manually obtained data from the hospital medical record database and from our department’s anaesthesiological database of all patients defined as palliative patients. These data included demographics, administrative information, anaesthesiological and procedural information, treatments received, and preprocedural symptoms. With regard to the symptoms, symptom categories were created, e.g. constipation, nausea, emesis, and jaundice were grouped under gastrointestinal; dyspnoea, pleural effusion, coughing under pulmonary symptoms. Symptoms were analysed when mentioned in the admission letters. To compare the study population to the total population of patients under anaesthesiological care in 2009, we obtained data on the total population that could be electronically extracted from the databases. This included age, sex, American Society of Anaesthesiologists (ASA) classification, diagnoses at discharge, hospital mortality, and priority of procedure.

### Data analysis

Data tabulation was performed using Microsoft Excel 2010 (Microsoft, Redmond, WA). The study population and the total population were compared using Pearson’s chi-squared test for nominal and ordinal variables and *t* test for metric variables. All *P* values were two-sided, and *P* values less than 0.05 were considered statistically significant. All analyses were carried out using IBM SPSS Statistics 21 (IBM, Armonk, NY).

## Results

**Fig. 1:**
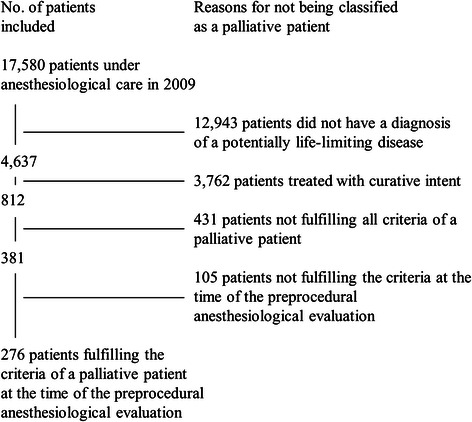
Make-up of the study population

Of 17,580 patients under anaesthesiological care at our hospital in 2009, 276 could be classified as palliative patients (1.57 %) (Fig. 1). The palliative patients were older, had a higher ASA-classification, showed more often the presence of an oncological disease, and had a higher hospital mortality than the non-palliative patients (Table 2). All these differences were statistically significant (*P* < 0.001). Concerning the oncological disease, the primary tumor site in most patients was the gastrointestinal tract (27.9 %) followed by the head/neck region (16.6 %) and tumors of the lung (8.7 %). Anaesthesiological and procedural parameters of the palliative patients are shown in Table 3. Most contacts with palliative patients occurred in the operating room (68.5 %). In a total of seven cases palliative patients were treated in a resuscitation situation, since anaesthesiologists lead the cardiac-arrest-teams in our hospital. In comparison to the non-palliative patients, procedures in palliative patients were significantly more often urgent or emergency procedures. Patients undergoing emergency procedures had a significantly higher in-hospital mortality than non-emergency patients (33.3 % vs 14.8 %, *P* < 0.001) and a significantly higher rate of treatment in the ICU (45.0 % vs. 31.0 %, *P* = 0.047).

**Table 2:** Demographic characteristics of patients with comparison

Number of patients, N	Patients classified as palliative (*N* = 276)	Patients not classified as palliative (*N* = 17,304)	*P* Value
Age in years (mean ± SD)	62.5 ± 12.0	52.5 ± 22.1	<0.001
Sex, N (%)			0.870
Women	105 (38.0)	6,500 (37.6 %)	
Men	171 (62.0)	10,804 (62.4 %)	
ASA-Status, N (%)			<0.001
I	4 (1.4)	3,493 (20.2)	
II	26 (9.4)	5,454 (31.5)	
III	198 (71.7)	6,636 (38.3)	
IV	40 (14.5)	1,505 (8.7)	
V	8 (2.9)	181 (1.0)	
VI	0 (0.0)	35 (0.2)	
Oncological disease, N (%)	269 (97.5)	4,513 (26.1)	<0.001
Hospital mortality, N (%)	52 (18.8)	858 (5.0)	<0.001

*SD* Standard Deviation, *IQR* Interquartile Range, *ASA* American Society of Anesthesiologists, *ICU* Intensive Care Unit

**Table 3:** Anesthesiological and procedural parameters of patients classified as palliative (*N* = 276)

Type of procedure, N (%)	
Operation	189 (68.5)
Intervention	64 (23.2)
Diagnostic	16 (5.8)
Cardio-Pulmonary-Resuscitation	
Emergency room	4 (1.4)
In-hospital	3 (1.1)
Priority of procedure, N (%)	
Elective	168 (60.9)
Urgent	48 (17.4)
Emergency	60 (21.7)
Patients treated because of their underlying, life-limiting disease	267 (96.7)
Patients treated during night or on weekends, N (%)	84 (30.4)
Length of hospital stay in days (median; [IQR])	21; [9–32]
Patients admitted to the ICU, N (%)	94 (34.1)
Length of ICU stay of patients admitted to the ICU (median; [IQR])	1; [1–6.25]
Palliative status mentioned on the anesthesiological record form, N (%)	12 (4.3)
DNR status or advance directive mentioned on the anesthesiological record form, N (%)	2 (0.7)
Duration of procedure in minutes (median; [IQR])	60; [30–110]
Duration of anesthesiological care in minutes (median; [IQR])	110; [70–175]
Type of anesthesia, N (%)	
Sedation	40 (14.5)
General anesthesia	215 (77.9)
Regional anesthesia	5 (1.8)
General + regional anesthesia	16 (5.8)

*DNR* Do-not-resuscitate, *IQR* Interquartile range

**Fig. 2:**
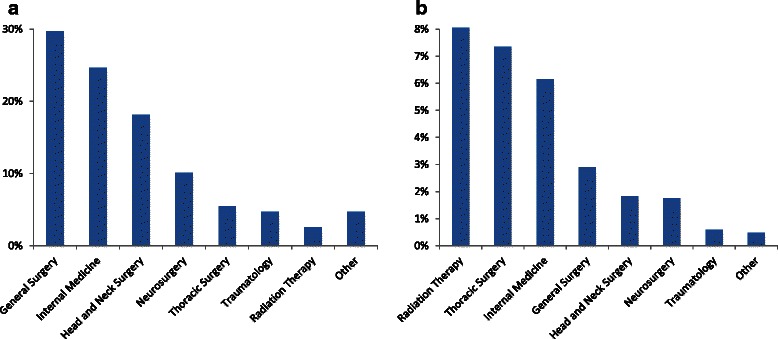
**a** Percentage of clinical specialties of palliative patients relative to the total number of palliative patients. (Other = clinical specialties with less than 5 palliative patients). **b** Percentage of palliative patients of a clinical specialty relative to the total number of patients under anaesthesiological care of that clinical specialty. (Other = clinical specialties with less than 5 palliative patients)

Of the 17,304 non-palliative patients, 12,621 (72.9 % vs. 60.9 % in palliative patients) were elective, 1,870 (10.8 % vs. 17.4 %) were urgent, and 2,813 (16.3 % vs. 21.7 %) were emergency cases (*P* < 0.001). Most procedures in palliative patients were directly related to the life-limiting disease; only 3.3 % had other reasons (e.g. myringotomy in a patient with otitis media, axillary-femoral bypass in a patient with lower limb ischemia). A substantial amount of palliative patients were treated after the regular working hours (7 a.m. to 4 p.m.) or on weekends (30.4 %). Palliative patients were rarely identified as palliative by the anaesthesiologist, who conducted the preprocedural interview and filled out the anaesthesia record (4.2 %). A do-not-resuscitate order was mentioned on only two anaesthesia records (0.7 %). Most palliative patients were treated by general surgery (29.7 %), internal medicine (24.6 %), and head and neck surgery (18.1 %) (Fig. 2a). In regard to the total number of patients under anaesthesiological care from one specialty (Fig. 2b), the highest rates of palliative patients were in radiation therapy (8.0 %), thoracic surgery (7.4 %), and internal medicine (6.1 %). Since receiving or being in need of a specific palliative therapy to alleviate symptoms was one of the criteria to define a patient as a palliative patient, all patients showed at least one symptom (range 1–6), with pain being the most prevalent symptom followed by gastrointestinal symptoms and nutritional problems (Table 4). 11.6 % of the palliative patients were seen by our hospital’s palliative care consultation service during their hospital stay, with 2.2 % being seen preprocedural.

**Table 4:** Symptoms of palliative patients at the time of anesthesiological evaluation

Symptoms/symptom group	Percentage of palliative patients showing symptoms
Pain	42.8 %
Gastrointestinal (e.g. nausea, constipation)	42.0 %
Nutritional (e.g. cachexia, swallowing disorder)	39.9 %
Pulmonary (e.g. pleural effusion)	34.4 %
Neuropsychiatric (e.g. depression, anxiety)	30.4 %
Airway (e.g. laryngeal obstruction)	16.7 %
Bleeding	9.4 %
Social (e.g. financial problems)	4.0 %
Other	12.3 %

## Discussion

Nearly 1 in 60 patients under anaesthesiological care in this university hospital could be classified as palliative. An important aspect of this article is how we define a palliative patient. While there is a definition for palliative care by the World Health Organization [14], there is no general consensus on how to define a palliative patient. We based the criteria of this study on the results of a systematic review by Van Mechelen et al. [13], who extracted the key elements for a definition of a palliative care patient. To interpret our study’s results, it is important not to treat “palliative” as equal to “non-curative”. There are numerous medical conditions, which cannot be cured but are stable and non-progressive, e.g. metastasized prostatic carcinoma in the elderly patient or chronic renal failure requiring dialysis. These patients would not require specialized palliative periprocedural treatment and were therefore excluded from the final analysis by our definition (Fig. 1). On the other hand, not all patients receiving palliative therapy were automatically included in the final analysis. Early palliative care becomes more and more available to patients; it is not restricted to patients with a far advanced disease anymore. Also we excluded all patients whose palliative status could not be recognized preprocedurally, because our study focuses on the anaesthesiologist’s assessment of the patient. For example a patient with a pancreatic tumour, that was seen to be irresectable intraoperatively, was not included in our analysis. If we had included these patients in our definition, we would have found a rate of 5.0 % palliative patients (875/17580 patients). It is difficult to compare our results to previous studies, because these studies investigated the rates of palliative procedures, resulting in rates in the range of 6 [12] to 12.5 % [11].

The comparability of our hospital mortality of 18.8 % in this patient population is limited by the fact that the other studies report 30-day or 90-day mortalities, which are 11 [12] and 6 % [15] respectively, again determined in patients undergoing palliative procedures. With the data available to us, it was not possible to determine survival rates or other mortalities than the hospital mortality.

Concerning the symptoms found, three out of the six most prevalent ones lie in the specific area of competence of an anaesthesiologist, pain, pulmonary symptoms, and airway problems. Also neuropsychiatric (e.g. delirium) and gastrointestinal (e.g. nausea and vomiting) symptoms are commonly seen and treated by anaesthesiologists postprocedurally. In the studied patient population anaesthesiologists could become more involved in the preprocedural stage and use their knowledge of symptom control to improve the patient’s well-being before the planned procedure and thereby taking over the role of a perioperative physician.

The results of our study also show, that palliative patients can be encountered in different and unexpected situations (e.g. as emergency cases, outside the operating room, after regular working hours). It is important for every anaesthesiologist to identify a patient as palliative and to recognize his medical problems to deliver optimum care. This seems to be either difficult or deemed as unimportant by the anaesthesiologist conducting the preprocedural interviews in our study, since only 4.3 % of the patients studied were classified as palliative on the anaesthesiological record. Identification of a patient as palliative could also lead to a higher involvement of a palliative consultation service, which has been shown to improve quality of care [16]. Palliative care in general should be initiated as early as possible [17]. We see the need for improvement at this point. The rate of patients seen by our hospital’s palliative consultation service is remarkably low, and patients are obviously seen too late. This indicates that the awareness for this patient group, and their potential benefit from specialised palliative therapy, is generally not high, also among the disciplines directly attending to these patients.

The importance of awareness for this patient group is underlined by the high number of patients undergoing invasive procedures at the end of life or at an advanced stage of disease [18, 19]. The number of these patients is likely to further increase in the future [20]. In this study we had seven patients undergoing cardiopulmonary resuscitation, showing that also in our patient population invasive procedures were undertaken.

Another point for improvement is the consideration of advance directives (AD) and do-not-resuscitate (DNR) orders in the periprocedural context. It is estimated that 25 % of oncological patients in Germany have formulated Ads [21], but these were only mentioned on 0.7 % anaesthesiological records of our patients. While the topic of ADs and DNR orders is widely covered in the international literature [8, 9], a discussion in Germany has yet to be started. There seems to be a low awareness of this problem, resulting in the low mentioning of ADs preprocedurally.

### Limitations

There are several limitations in our study. Since we employed a retrospective study design, we are depending on complete and correct data documentation. The patient classification was based on the discharge diagnosis, which is double checked before being entered into our hospitals database, because it is directly relevant for the financial revenue in the German hospital financing system. Therefore we assume a low rate of missing and incorrect data with this parameter. Other variables might not be so robust, probably the weakest parameter were the symptom groups. Most of them were derived out of the discharge letters and from other physician’s notes. Rates could well be higher than those mentioned in these documents. Also, we did not examine and evaluate the treatment by anaesthesiologists to provide symptom control. We wanted to show, that there are problems that should be considered during the perioperative period and lie in the scope of the field of anaesthesiology.

Another point to discuss are the criteria chosen to define a patient as palliative. Since widely accepted criteria are missing, we based our criteria on a systematic review on this topic [13]. We applied these criteria in a strict way, to be sure not to overestimate the rate of palliative patients.

Finally we conducted our study at a single institution. Our results cannot be seen as representative for other hospitals, especially because our hospital is missing certain departments that treat oncological patients such as gynaecology, paediatric surgery, and urology.

## Conclusions

This study shows that anaesthesiologists treat palliative patients under varying circumstances. Palliative patients show a high range of symptoms needing the attention of specialist medical treatment in the periprocedural period, which can be ideally provided by anaesthesiologists. Awareness for this patient group needs to be heightened to adequately recognize and treat these patients. One way to achieve this goal would be to implement a “palliative pathway” for these patients. This would include identifying the patient status by the primarily treating specialty as early as possible, mentioning this status on the notification for the anaesthesiological evaluation, thereby setting the focus on optimized symptom control and recognition of therapeutic restrictions such as AD and DNR orders by the anaesthesiological caregivers, and also integrating palliative care specialists for continuing good symptom control postprocedurally and after discharge from the hospital.
